# Epigenetic and Genetic Factors Associated With Opioid Use Disorder: Are These Relevant to African American Populations

**DOI:** 10.3389/fphar.2021.798362

**Published:** 2021-12-22

**Authors:** Christopher A. Blackwood, Jean Lud Cadet

**Affiliations:** Molecular Neuropsychiatry Research Branch, NIH/NIDA Intramural Research Program, Baltimore, MD, United States

**Keywords:** epigenetics, opioid use disorder (OUD), DNA methylation, mu opioid (MOP) receptor, African Americans

## Abstract

In the United States, the number of people suffering from opioid use disorder has skyrocketed in all populations. Nevertheless, observations of racial disparities amongst opioid overdose deaths have recently been described. Opioid use disorder is characterized by compulsive drug consumption followed by periods of withdrawal and recurrent relapses while patients are participating in treatment programs. Similar to other rewarding substances, exposure to opioid drugs is accompanied by epigenetic changes in the brain. In addition, genetic factors that are understudied in some racial groups may also impact the clinical manifestations of opioid use disorder. These studies are important because genetic factors and epigenetic alterations may also influence responses to pharmacological therapeutic approaches. Thus, this mini-review seeks to briefly summarize what is known about the genetic bases of opioid use disorder in African Americans.

## Introduction

Opioid overdose is one of the leading causes of deaths in the United States (U.S.) (Jalal et al., 2018). The over-prescription of opioids and the illicit use of these drugs are amongst the factors that have contributed to the rise in opioid overdoses and the development of opioid use disorder (OUD) (CDC, 2020; Blackwood and Cadet, 2021b; Mattson et al., 2021). OUD is a chronic relapsing neuropsychiatric disorder characterized by compulsive drug intake despite negative life consequences (DSM-V, 2013). Importantly, there are reported racial differences in people suffering from OUD (Terry et al., 2008; Nielsen et al., 2010; Xu et al., 2018). In addition, certain African American populations are reported to be dying from opioid overdoses at higher rates than other ethnic groups (Carlesso and Kara, 2019; Chau, 2020; DeLaquil, 2020; Furr-Holden et al., 2021). It is therefore important to understand the potential causes for these observations of differential overdose rates for prevention purposes.

Opioid drugs have their biological effects by stimulating opioid receptors that are members of the G protein coupled receptor (GPCR) family (Shippenberg et al., 2008; Pasternak and Pan, 2013; Gendron et al., 2016). These receptors include mu(μ)-, delta(Δ)-, and kappa(Κ)-opioid receptors that can form homo- and heterodimeric complexes and transduce intercellular signals through various cellular pathways (Fujita et al., 2014; Bruchas and Roth, 2016). Among these, the μ-opioid receptor appears to be more relevant to addictive processes (Bossert et al., 2018; Blackwood et al., 2019) and is the main target for FDA-approved drugs used to treat OUD (Schuckit, 2016).

The μ-opioid receptor is encoded by the OPRM1 gene (Chen et al., 1993; Thompson et al., 1993). The receptor is expressed throughout the brain (Hirvonen et al., 2009; Johansson et al., 2019) and has high affinity to endogenous opiates including ß-endorphin (Zadina et al., 1997). Stimulation of μ-opioid receptor is responsible, in part, for opioid-induced euphoria and rewarding effects (Wang, 2019). Repeated stimulation of the μ-opioid receptor is also mainly responsible for the development of tolerance and physical dependence on opioid drugs (Kieffer and Evans, 2002). In clinical settings, various pain syndromes are treated with μ-opioid receptor agonists (Basbaum and Fields, 1984; Pasternak, 1993). Therefore, factors that interfere with the pharmacokinetics and signaling of the μ-opioid receptor could impact the treatment of pains syndrome and/or the manifestation of OUD in various ethnic populations.

Here, we provide a short review of potential genetic and epigenetic factors that impact the μ-opioid receptor gene. These include mutations reported in the μ-opioid receptor gene and some epigenetic factors including DNA methylation and transgenerational epigenetic inheritance that might impact gene expression in the brain. Finally, we consider the possibility that these might impact clinical course of OUD in African American populations.

## Single Nucleotide Polymorphisms

Genetic variations due to single nucleotide polymorphisms (SNPs) are factors that can influence susceptibility to substance use disorders (SUDs) (Oslin et al., 2003; Haerian and Haerian, 2013)**.** Most SNPs are not necessarily associated with any discernible functional changes in mRNA expression or protein functions. However, some SNPs can result in amino-acid substitutions that change the functionality of proteins that can result in susceptibility to brain diseases or adverse consequences during exposure to stressful environmental stimuli (Shi et al., 2011; Lee et al., 2017)**.**

## SNPs Identified in the μ-Opioid Receptor Gene

Several groups of investigators have reported on potential links between OUD and SNPs identified in the human μ-opioid receptor gene (Bond et al., 1998; Zhang et al., 2005). These SNPs include C17T, G24A, G799A, G942A, and A118G (Bond et al., 1998), with the A118G variant being the most commonly reported. This SNP is located at position 118 (A118G) and corresponds to an amino-acid conversion from asparagine to aspartate at position 40 of the N terminus site of the receptor protein (Bond et al., 1998). Interestingly, Bond et al. (1998), reported that the A118G variant showed greater affinity for ß-endorphin. In addition, activation of the A118G variant is much more potent at the G-protein-coupled protein potassium ion channels than the normal variant (Bond et al., 1998). Of further clinical relevance, the A118G variant is an important mediator of endocytosis and desensitization of the human μ-opioid receptor (Beyer et al., 2004), implicating it in the development of greater tolerance in individual patients who use or misuse opioid drugs. Postmortem tissues that have revealed decreased mRNA expression and protein levels of the human μ-opioid receptor in humans with the A118G variant (Zhang et al., 2005) further implicate them in the clinical course of OUD patients.

## Potential Implications of A118G in African American Patients With OUD

It has been reported that the occurrence of the A118G variant may vary across ethnic groups (Bond et al., 1998; Hastie et al., 2012; Abijo et al., 2020). Specifically, allele frequencies of the A118G variant have been investigated in healthy and opioid-exposed African Americans (Bond et al., 1998; Hastie et al., 2012). Bond et al. (1998) used heroin-exposed and non-drug exposed subjects found an overall allelic frequency (A/G and G/G) of 3.3% in African Americans compared to 21.2 and 25.4% in Whites and Hispanics, respectively. A subsequent study by Hastie et al. (2012) who included healthy young adults in their paper documented that the frequency of the A118G variant was less common in African Americans (7.4%) compared to Whites (28.7%) and Hispanics (27.8%). The presence of A118G polymorphisms in African Americans and other ethnic groups might affect the binding affinity of the μ-opioid receptor. The changes in binding affinity might hamper pain perception, reduce response to analgesic drugs, and increase self-administration of opioid drugs (Beyer et al., 2004; Janicki et al., 2006; Sia et al., 2008).

A118G polymorphism removes a highly conserved N-glycosylation site in protein’s extracellular domain (Beyer et al., 2004) that may hamper pain perception in chronic diseases (Janicki et al., 2006), reduce response towards analgesic drugs (Oertel et al., 2006), and tend to increase administration of opioids (Sia et al., 2008).

## DNA Methylation in Eukaryotic Cells

**FIGURE 1:**
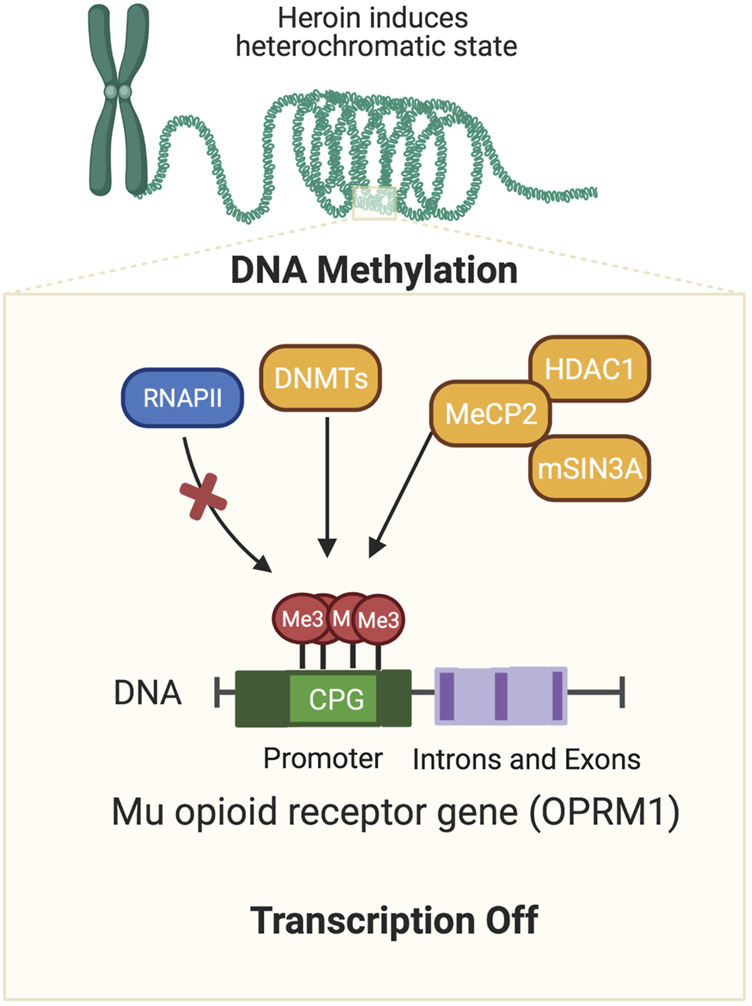
Opioid-induced DNA methylation of mu opioid receptor gene. Agents such as opioids have been shown to cause epigenetic alterations in the brain. This cartoon illustrates how exposure to a mu opioid agonist can cause hypermethylation at CPG sites located in the promoter regions of the mu opioid receptor gene. DNA methylation is followed by the recruitment of co-repressors including HDAC1, DNMTs, and/or MBD proteins (MeCP2). Together these protein form complexes that can repress gene transcription repression by preventing binding of transcription factors on the DNA promoters or relevant transcription sites of relevant genes.

Repeated exposure to drugs that lead to SUD in some individuals is related, in part, to neuroadaptive epigenetic alterations that occur in the brains of exposed individuals (Robison and Nestler, 2011; Cadet, 2016; Cadet and Jayanthi, 2021). Epigenetic events occur through DNA methylation, chromatin remodeling, non-coding RNA, and histone modifications (Robison and Nestler, 2011). The next paragraphs focus on the role of DNA methylation in OUD because DNA methylation plays an important role in the transcription of the μ-opioid receptor gene (Figure 1) (Hwang et al., 2007; Chidambaran et al., 2017).

DNA methylation refers to the addition of methyl groups to cytosine residue. This reaction is catalyzed by the enzymes, DNA methyltransferases (DNMTs) (Kinney and Pradhan, 2011; Smith and Meissner, 2013). Changes in DNA methylation can occur in enhancers and promoters of genes (Kinney and Pradhan, 2011; Smith and Meissner, 2013). DNA methylation is required for normal development in humans (Kim et al., 2009; Smith and Meissner, 2013). Changes in DNA methylation may lead to repression of gene expression through the recruitment of histone deacetylases (HDACs), histone methyl/lysine-transferases (HMATs/KMTs), as well as DNA methyl-binding domain proteins (MBDs) methyl-CpG binding protein 2 (MeCP2) (Hyun et al., 2017; Voss and Thomas, 2018). Alterations in DNA methylation are influenced by environmental stress and chemical exposure**.** DNA methylation plays important roles in the pathobiology of cancer and neurodevelopmental disorders (Robertson and Wolffe, 2000).

## DNA Methylation and μ-Opioid Receptors

Alterations in DNA methylation have been reported after chronic exposure to several rewarding drugs including opioids (Hwang et al., 2007; Host et al., 2011; Manzardo et al., 2012; Pol Bodetto et al., 2013; Chidambaran et al., 2017; Jayanthi et al., 2020). Specifically, Hwang et al. (2007) had reported that increased DNA methylation of CpG sites in the promoter regions of the μ-opioid receptor gene led to increased binding of MeCP2 followed by recruitment of the repressors, histone deacetylase 1 (HDAC1) and SIN3 transcription regulator family member A (mSin3A), thus leading to downregulation of the μ-opioid receptor gene. Their findings and interpretation are consistent with observations that MeCP2 can negatively regulate the expression of the μ-opioid receptor gene (Lu et al., 2009; Garcia-Concejo et al., 2016).

## DNA Methylation and Opioid Use Disorder

Aberrant alterations in DNA methylation have been reported to be associated with various neuropsychiatric diseases including heroin use disorder (Nielsen et al., 2008; Cadet, 2016; Wang et al., 2016; Cadet and Jayanthi, 2021). For example, Nielsen et al. (2008) reported that DNA taken from lymphocytes of patients suffering from heroin use disorder showed increased levels of DNA methylation. Similarly, Xu et al. (2018) reported DNA hypermethylation in former patients suffering from heroin use disorder compared to healthy controls who had no history of opioid consumption. The results of these two papers are consistent with those of other investigators who have found that patients suffering from heroin use disorder showed altered DNA methylation at CpG sites located in the promoter region of the μ-opioid receptor (Chorbov et al., 2011; Doehring et al., 2013; Ebrahimi et al., 2018). Specifically, Chorbov et al. (2011) reported that former patients suffering from heroin use disorder stabilized in methadone treatment showed increased DNA methylation in two CPG sites at +182 and +186 loci of the promoted of the μ-opioid receptor. In addition, Doehring et al. (2013) documented increased DNA methylation in one CpG site at +136 loci of the receptor promoter in heroin-dependent patients and in opioid-treated pain patients in comparison to the non-opioid exposed individuals (Doehring et al., 2013). Taken together, these results implicate a role of DNA methylation of the opioid receptor gene in OUD.

## DNA Methylation and African Americans

Although limited, it has been suggested that there are differential changes in DNA methylation levels across individuals from various racial groups exposed to opioids (Nielsen et al., 2009; Nielsen et al., 2010). Nielsen et al. (2009) had initially reported that hypermethylation at CpG sites located in the promoter of the μ-opioid receptor was linked to long-term heroin consumption. Subsequently, they observed that African American individual patients showed higher levels of DNA methylation upstream of the promoter region of the μ-opioid receptor in comparison to Hispanic or White patients (Nielsen et al., 2010). These observations need to be replicated in much larger patient populations before any rigorous interpretations can be made with confidence.

## Intergenerational and Transgenerational Epigenetic Inheritance

**FIGURE 2:**
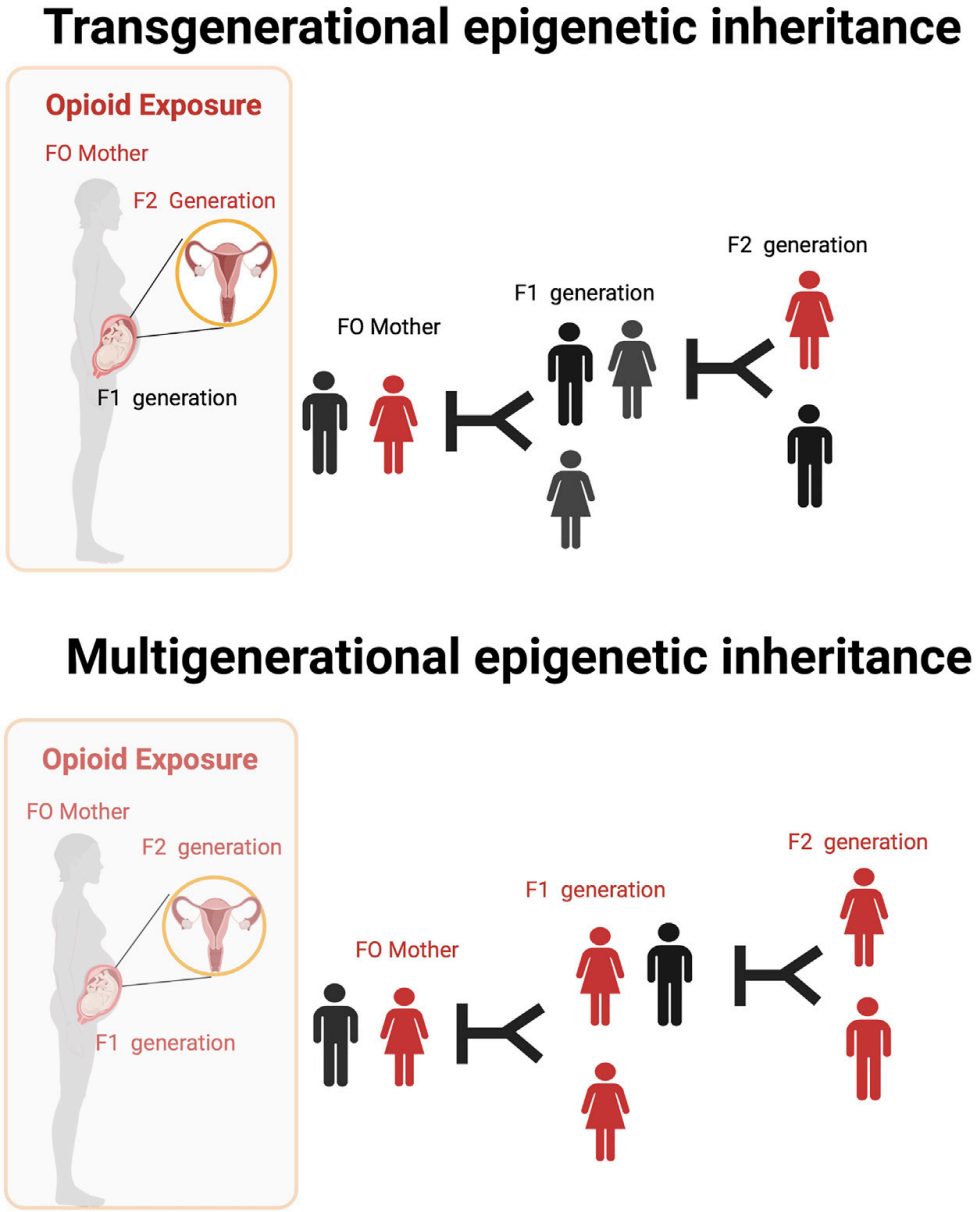
Transgenerational and Multigenerational epigenetic inheritance of opioid exposure. The ability to transmit epigenetic changes from one generation to the next in experiments done in animal models. There is very little direct evidence of similar occurrences in humans. Opioid use by mothers (or fathers) might lead to permanent changes in the DNA found in their ovaries (or sperm) that are transmissible to later generations even in the absent of direct drug exposure (transgenerational epigenetic inheritance). Multigenerational epigenetic inheritance coincident direct exposure of multiple generations to an environmental factors promoting alterations in the multiple generations exposed.

Opioid exposure is thought to be associated with changes in a germline, which may be transmitted to subsequent generations in a process called intergenerational or transgenerational epigenetic inheritance (Cicero et al., 1995; Sarkaki et al., 2008; Byrnes et al., 2012; Byrnes et al., 2013; Vassoler et al., 2017; Toorie et al., 2021). Intergenerational and transgenerational epigenetic inheritance refers to phenotypic variation that does not stem from variations in DNA base sequences that are transmitted through the germline to the immediate offspring even in the absence of direct opioid exposure (Vassoler et al., 2014; Odegaard et al., 2020). Some of these ideas are illustrated in Figure 2.

In an interesting study, adolescent female rats injected with increasing dosages of morphine were mated with drug-free males (F0 animals) (Byrnes et al., 2013). The F1 and F2 offsprings that derived from the F0 female were then investigated for locomotor defects after injection with a dopamine D2 receptor (D2R) activator, quinpirole. Repeated administration of quinpirole in the F1 and F2 progenies reduced locomotor sensitization in activity testing chambers (Byrnes et al., 2013). In the same study, the F1 and F2 progenies showed increased expression of D2R and Κ-opioid receptors in the nucleus accumbens. Using a similar model with morphine, F1 offspring from the morphine-exposed female showed decreased anxiety-like behavior in open field activity experiments and increased sensitivity to opioid rewarding effects (Byrnes et al., 2011).

In another interesting study, performed by Vassoler et al. (2017), females adolescent rats exposed to morphine were bred with drug-naïve males. In this study, F1 and F2 offsprings from the maternal line were found to have lower levels of morphine self-administration and reduced relapse-like behavior. Additionally, they showed altered expression of genes associated with synaptic plasticity in the nucleus accumbens (Vassoler et al., 2017). A study performed by Odegaard et al. (2020) found that pregnant mothers exposed to oxycodone showed developmental impairments that were displayed in multiple generations. These findings illustrated the potential transgenerational and multigenerational influences of opioids exposure in females.

## General Summary and Conclusion

The prevalence of OUD and its associated consequences including overdose deaths have increased in recent years. Unfortunately, African Americans have been reported to have suffered some of the largest increases in opioid-related overdose deaths (Patel et al., 2021). The potential ramifications of these changes in the course of the COVID-19 infection has also been discussed (Blackwood and Cadet, 2021a). Some of these complications may be related to the lack of access of African American individual patients to psychiatric care that has been shown to be an area of major racial inequities (Hall et al., 2021; Stahler et al., 2021). This discussion suggests the need to increase available resources to increase access to treatment programs by African Americans who seem to be suffering from the brunt of the disasters associated with the opioid epidemic in the United States.

It needs to be further commented that more expansive genetic and epigenetic studies are needed in order to compare individual and group racial differences in the susceptibility to or resistance against OUD. This statement is based on some initial studies that have reported differences in genetic and epigenetic markers across various ethnic groups (Nielsen et al., 2010; Abijo et al., 2020). For example, it will be important to investigate the potential connections between the A118G variant and the clinical course of OUD in populations that include large numbers of patients and non-patients from various ethnic American groups. So far, it is very obvious that most of these genetic studies have focused on white populations and have neglected the African American communities in the United States. Without engaging these populations, the racial inequities in diagnosis, treatment, and mortality will continue to rise. Similarly, the initial findings of differences in DNA methylation of the opioid receptor gene need to be investigated further in similar large population of controls and patients from various ethnic groups. Without these studies, it is possible that prevention approaches that treat all populations based on findings in white populations will fail. The suggested approaches are also relevant to the practice of precision medicine that will take the treatment of SUD into the next decades.
